# Pathogenicity Traits Correlate With the Susceptible *Vitis vinifera* Leaf Physiology Transition in the Biotroph Fungus *Erysiphe necator*: An Adaptation to Plant Ontogenic Resistance

**DOI:** 10.3389/fpls.2018.01808

**Published:** 2018-12-11

**Authors:** Agnes Calonnec, Jerome Jolivet, Philippe Vivin, Sylvain Schnee

**Affiliations:** ^1^UMR1065 SAVE Santé et Agroecologie du Vignoble, INRA, ISVV, Villenave d'Ornon, France; ^2^EGFV, Bordeaux Sciences Agro, INRA, University of Bordeaux, y, Villenave d'Ornon, France

**Keywords:** powdery mildew, ontogenic resistance, sink-source transition, traits of pathogenicity, grapevine

## Abstract

How and when the pathogen cycle is disrupted during plant development is crucial for harnessing ontogenic resistance in sustainable agriculture. Ontogenic resistance against powdery mildew (*Erysiphe necator)* was quantified on *Vitis vinifera*. Shoots were sampled in the vineyard at several dates during seasonal growth and processed in the laboratory under controlled conditions. Experiments were conducted on two susceptible *Vitis vinifera* Cabernet Sauvignon and Merlot. The process of leaf ontogenic resistance was investigated by measuring three quantitative traits of pathogenicity: the infection efficiency, sporulation and mycelium growth. Morphological and physiological plant indicators were used to identify leaf changes that resulted in ontogenic resistance and to predict pathogen variations that were linked to pathogenicity traits. The process of ontogenic resistance was established early in correspondence with the physiological transition of the leaf from sink to source status and was characterized by its increase in sugar content. The three traits of pathogenicity that we measured were affected, and their variation was strongly correlated with leaf age. Using leaf age, we were able to accurately predict the susceptibility of the leaf: a leaf aged, on average, 13.3 days had a very high probability (0.8) of being susceptible, while this probability decreased to 0.5 one week later. Sporulation was more closely correlated with variations in sugar and the infection efficiency in leaf water. The results for both cultivars were consistent. Ontogenic resistance on grapevine leaves is thus interpreted to be a strong, immutable physiological process that *E. necator* is able to circumvent by restricting its development to sink tissue. Future research should explore how this native plant resistance can be incorporated into grape management strategies to better control powdery mildew (PM) epidemics with reduced amounts of fungicides.

## Introduction

Biotrophs totally depend on their hosts to complete their life cycles, deriving their nutrients from living host cells through the development of specialized infection structures (O'Connell and Panstruga, 2006). *Erysiphe necator* Schwein, the powdery mildew of grapevines, is very efficient and can adopt highly sophisticated mechanisms to invade living plant cells successfully, such as upregulating the expression of cell wall invertase and plant hexose transporter genes or downregulating vacuolar invertase genes (Hayes et al., 2010).

Powdery mildew of grapevine is a ubiquitous disease that affects common cultivars of *Vitis vinifera* L., and it is responsible for significant damage (Calonnec et al., 2004) if no protective measures are used. While fungicide-based strategies are generally effective in reducing disease (Delière et al., 2010; Deliere et al., 2015; Valdés-Gómez et al., 2017), finding alternative methods that consider practitioners and the environment and that are economically feasible remains a challenge for sustainable viticulture. One solution relies on the exploitation of resistant grape varieties, in which sources of resistance coming from other *Vitis* of American or Asian origin are introgressed (Calonnec et al., 2013b). The difficulty for breeders in associating traits of agronomical and oenological values and genes of pathogen resistance leads also to exploration of the native potential of plant defenses in cases of a susceptible *V. vinifera* host. Innovative strategies to counteract pathogen efficiency could rely on integration into the management of the disease of the time-course evolution of the plant (i.e., leaves and berries) resistance during the season (Burie et al., 2011; Valdes-Gomez et al., 2011; Gadoury et al., 2012; Calonnec et al., 2013a).

In many plant-pathogen interactions, a susceptible host can acquire resistance to a pathogen at a certain developmental stage (Develey-Rivière and Galiana, 2007). This age-related resistance, commonly called ontogenic resistance (when young tissues are susceptible) or receptivity (when old tissues are susceptible), could be defined as the dynamic modification of tissue receptivity during organ development, triggering resistance/tolerance to pathogenic micro-organisms. Ontogenic resistance has been described for many plant-pathogen systems [e.g., strawberry-powdery mildew (Carisse and Bouchard, 2010), cucurbit fruit—*Phytophthora capsici* (Ando et al., 2009), tobacco—*Phytophthora parasitica* (Hugot et al., 1999), apple-apple scab (Li and Xu, 2002), cocoa—*Phytophthora megakarya* (Takam Soh et al., 2012), pea-powdery mildew (Fondevilla et al., 2006), pea-aschochyta blight (Richard et al., 2012)]. They are therefore common traits but remain underexploited in disease management, principally because of a lack of understanding of the underlying mechanisms and of the potential variability linked to the physiological responses of the host to environmental factors. The phenomenon of ontogenic resistance or receptivity to the main pathogens of grapevines has been highlighted and assessed on bunches for *Plasmopara viticola* (Kennelly et al., 2005), *Guignardia bidwellii* (Molitor and Berkelmann-Loehnertz, 2011), and *Botrytis cinerea* (Deytieux-Belleau et al., 2009). Botrytis susceptibility is known to increase with fruit maturity. Although the mechanisms involved remain speculative, they might differ from those involved in response to infection in the classical defense system (Develey-Rivière and Galiana, 2007; Gee et al., 2008). For powdery mildew, the process has been underscored for berries, on which infection stops when berry sugar content reaches 8% (Delp, 1954), and it is characterized by a low penetration rate of infectious structures following potential modification of the structure and chemical composition of the cuticle (Ficke et al., 2002). The level of irradiance received before inoculation can strengthen ontogenic resistance through modifications of berry physiology, such as decreases in pH and K concentration and increases in polyphenol and anthocyanin concentrations (Zahavi and Reuveni, 2012). Ontogenic resistance also exists in mature leaves, which suffer less infection (Doster and Schnathorst, 1985). Experiments on cuttings grown in a glasshouse under a controlled temperature showed that infection is maximal for leaves that have just ceased importing assimilates (Merry et al., 2013). Simulations have shown that, depending on its parameter value, ontogenic resistance could significantly reduce the severity of late epidemics (Calonnec et al., 2008, 2011). However, little information is available regarding the behavior of the pathogen at each step of its sequential development for a broad range of crop development, and no information is available for field-grown material.

In our study, leaf ontogenic resistance to powdery mildew in *V. vinifera* was investigated using the susceptible cultivars Cabernet Sauvignon and Merlot. They are the two most commonly grown cultivars in the world for wine grapes (OIV, 2018). Shoots were characterized and sampled in the field, and leaves were processed in the laboratory. Our questions were: When is ontogenic resistance established? How does it vary with variety, crop development and climatic environment? Which pathogenicity traits are the most affected, and how variable is the effect? We used leaf age, leaf surface, sugars and LWC to identify changes in leaf physiology linked to ontogenic resistance and to determine whether they could be used to predict variations in pathogenicity.

## Materials and Methods

### Plants

*Vitis vinifera* Cabernet cv. Sauvignon and Merlot were grown on two INRA experimental vineyards near Bordeaux, France, at Cadaujac and Latresne, respectively. Plantation density was 5,050 vines/10,000 m2; the vinestocks were between 7 and 12 years old. The experimental plots were regularly protected with fungicides from natural infection by downy (“Remiltine or Dauphin” (cymoxanil, mancozeb) and powdery mildews (powder or wettable sulfur). Treatments were applied between 6 and 20 days before sampling. Plant phenological stages were monitored from bud break until ripening (Baillod and Baggiolini, 1993). Each new fully expanded primary leaf (width from 3 cm) was tagged by a set of plastic color markers to indicate the date of leaf appearance and subsequently to calculate leaf age. These color markers allowed leaf age (LA) and the sum of temperatures (above 10°C) accumulated by the leaf during its development (LAdev) to be determined.

### Bioassays

Shoots were sampled at different dates during the vegetative growth of grapevines (May 12, May 26 and June 10 for Cabernet Sauvignon) in 2005; June 4, 2009, and May 27 and June 24, 2010, for Merlot). Selected shoots were cut early in the morning and immediately brought to the laboratory, with the base of the shoots kept in water. No more than one shoot per vine. Before leaf sampling, petioles were marked with a color code to specify their respective positions on the shoot and to maintain traceability throughout the whole experiment. An approximation of the leaf extension rate (LER) per day was calculated by measuring leaf dimensions 3 days (t-3) and the day before (t-1) sampling with. $\text{LER}=\frac{\left({\text{LS}}_{\text{t-3}}-{\text{LS}}_{\text{t-1}}\right)/2}{\left({\text{LS}}_{\text{t-3}}-{\text{LS}}_{\text{t-1}}\right)/2}$The maximum leaf width (W) and maximum leaf length (L) of each leaf were measured to calculate the leaf surface (LS) in accordance with Montero et al. (2000) (LS = L × W × 0.6). For each shoot, the leaves were detached, abundantly washed under permuted water and then dried on filter paper. Under axenic conditions, the leaves were disinfected in a 5% (w/v) aqueous calcium hypochlorite at 65% for 10 min, rinsed in sterile water and dried in sterile filter paper. Three leaf discs (Ø 22 mm) were cut into each leaf and distributed in three different Petri dishes prepared for the pathogenicity tests. The leaf discs were disposed abaxial face down on an agar medium (20 g L^−1^) supplemented with benzimidazole (30 mg L^−1^). Each Petri dish contained six discs from an identical leaf level (not necessarily the same LA, as shoots are not synchronous in their growth) from six different shoots (replicates). Petri dishes corresponding to the infection efficiency test and to the total production of spores per cm2 were randomly placed in a settling tower and then artificially inoculated by blowing conidia from a leaf infected 14 days earlier (4–5 conidia on each conidiophore), in accordance with Cartolaro and Steva (1990). All leaf levels were inoculated together in one tower. Two towers were necessary to inoculate all of the replicates for each experiment. The average density of spores inoculated was 120, 388, and 610 spores/cm2 for 2005, 2009, and 2010, respectively. The dishes were placed in a growth chamber at 22°C (12:12 h light:dark photoperiod) for pathogen development. One different monospore isolate was used each year. It was sampled from vinestock leaves from a vineyard in the Bordeaux area or in the greenhouse before each annual experiment and was bulked on Cabernet Sauvignon leaves from greenhouse-grown cuttings. Infection efficiency was assessed at 72 hpi (hours post-infection) by counting the conidia that reached the branching hyphae stage (stage 5–6 according to Leinhos et al., 1997) after removing tape from leaves and then applying a cotton blue staining procedure (Cartolaro and Steva, 1990). The total production of spores per infected disc was measured at 14–15 dpi. For colony growth and spores per colony tests, the leaf discs were inoculated by the deposit, with a needle, of a few conidia (from 1 to 10) in their centers. Colony growth was assessed at ~4, 7, 10, 14, and 18 dpi (days post-infection) and the production of spores per colony at 14 or 18 dpi. Sporulating disks were placed in a vial filled with 20 mL of isotone 2 and one drop of non-ionic dispersant (Nacconol *90F*), shaken, and the spore production was assessed by counting the number of particles between 15 and 38 μm diameter on a sample of 500 μl using a particle counter (Coulter Counter® Multisizer^TM^ 3—Beckman Coulter, U.S). The disease variables calculated were: Inf (% of infectious spores as defined above), Spo (number of spores per cm^2^ of infected leaf disc), Spoc (number of spores per isolated colony), and Diam (final colony diameter in mm). A total of 12–20 shoots (120–220 leaves) were tested depending on the experiments. The experimental conditions and variables measured are summarized in Tables 1, 2, respectively and experimental design in Supplementary Figure S1.

**Table 1:** Conditions of the experimentations.

					**Climatic conditions before the experiment**						
				**Phenological stage**	**During the 10 days before D**				**at D-1**	**at D-1**	
**Experiments**	**Variety**	**Date of sampling = D**	**Nb sampled shoots**	**mean Nb leaf/shoot ±σ**	**Average T^**°**^C**	**GD**	**Sum Rain mm**	**Average ETPP**	**Average global radiance J.cm^**2**^**	**GD**	**Average T^**°**^C**
Exp. 1	Cabernet Sauvignon	12-May	20	6.05 ± 0.74	15.2	51.9	1.5	3.82	2481	212.4	16.8
Exp. 2	Cabernet Sauvignon	26-May	20	9.5 ± 1.43	16.2	61.6	0.5	4.34	2868	295.7	20.7
Exp. 3	Cabernet Sauvignon	9-Jun	15	14.73 ± 1.34	18.3	83	0.5	4.9	3049	423.7	19.1
Exp. 4	Merlot	4-Jun	12	14.33 ± 1.37	18.7	86.9	11	3.78	4677	323.5	22.3
Exp. 5	Merlot	27-May	6	12.50 ± 0.96	18.1	84.2	5	4.03	2290	271	17.6
Exp. 6	Merlot	24-Jun	6	20.17 ± 1.10	15.9	58.9	48	3.09	4792	474.3	19.4

*Nb, number; σ, standard deviation; ETPP, Evapotranspiration rate; GD, growing degrees, sum of degrees above 10°C*.

**Table 2:** Measured variables for the experiments.

**Variables**	**Abbr**.	**Units**	**Experiments**					
			**Exp. 1**	**Exp. 2**	**Exp. 3**^a^	**Exp. 4**	**Exp. 5**	**Exp. 6**
**PLANT GROWTH VARIABLES**								
Leaf age	LA	Day	•	•	•	•	•	•
Leaf temperature of development	LAdev	Sum of °C days above 10	•	•	•	•	•	•
Leaf surface	LS	cm2	•	•	•	•	•	•
Leaf extension rate	LER	Day^−1^	•	•	•	•	•	•
**PHYSIOLOGICAL INDICATORS**								
Soluble sugars	SS	(glucose + fructose + saccharose) in % of dry weight	•	•		•	•	•
Complex sugars	CS	Starch expressed in equivalent glucose in % of dry weight	•	•		•	•	•
Leaf water content	LWC	–	•	•		•	•	•
**DISEASE**								
Infection efficiency	Inf	Rate of branched conidia at 72 hpi	•	•	•	•	•	•
Relative infection	RInf	Relative rate	•	•	•	•	•	•
Maximal colony diameter	Diam	mm	•	•	•	•	•	•
Sporulation per colony	Spoc	Number of spores generated per colony	•	•	•	•	•	•
Sporulation per leaf area	Spo	Number of spores generated per cm2 of inoculated leaf area	•	•	•	•	•	•
Relative sporulation	RSpo	Relative rate	•	•	•	•	•	•

a *No physiological indicators available*.

### Physiological Indicators

For each sampled leaf, six discs (Ø 6 mm) were collected into a 2-ml Eppendorf tube, fresh weighed, dried in a hood (60–70°C for 72 h) and then weighed again. Leaf water content (LWC) was calculated from fresh (FW) and dry mass measurements (DW) (LWC = (FW − DW)/FW). The total non-structural carbohydrates were extracted and dosed as described by Gomez et al. (2007). Briefly, two metallic balls (Ø 5 mm) were added to each disc leaf samples, and the Eppendorf tube was submitted to intense shaking (3 min at 30 swings sec-1, Retsch Mixer Mill MM200). An ethanolic solution (80%, v/v) was added to the finely ground tissue, sonicated for 10 min at 60°C and centrifuged (3,700 trs min^−1^ for 5 min). The pellet containing soluble starch (ST) was rinsed three times with nanopure water, autoclaved and then hydrolysed in the presence of 200 μl of amyloglucosidase at 55°C for 1 h. The supernatant representing soluble sugars (including glucose, fructose and sucrose) (SS) was dried and then hydrolysed by addition of 200 μl of invertase (Sigma 19253) at 55°C for 1 h. After drying, 800 μl of nanopure water were added to the two types of extracts and vigorously shaken by vortex agitation and sonication to ensure total carbohydrate solubilisation. Soluble sugars and starch solutions were prepared (kit LISA 200C, CETIM, France) and measured spectrophotometrically at 340 nm, with an ELx800UV automated micro-plate reader (Biotek Instruments Inc., Vermont, USA). Glucose (Sigma G8270) was used for the standard range, and all of the obtained data were expressed in glucose equivalents.

### Environmental Growing Conditions

The sum of degree-days above 10°C (growing degrees) was used to determine grapevine organogenesis (Schultz, 1992). Five variables were calculated to characterize the environmental conditions of vine growth: the sum of GD at the day of sampling, which is also an indicator of the time of the experiment; the sum of GD during the 10 days before sampling; the average evapotranspiration rate, according to the Penman formula for this period (ETPP in mm.day^−1^); and the sum of rainfall for the 10 days before the experiments. The environmental conditions are summarized in Table 1.

### Data and Statistical Analyses

For infection efficiency (Inf), only data based on more than 30 counted spores were analyzed, and for all variables, only shoots with more than 3 validated samples were considered. To lessen the variation between shoots and to emphasize the variation between leaves of different ages, relative infection (RInf) and sporulation (RSpo) rates were also calculated by dividing the value obtained for a given leaf by the maximum value on its own shoot.

Variance analyses (general linear modeling procedure in SAS) were performed on different variables, such as infection efficiency (Inf), sporulation (Spo), maximum diameter (Diam), and the number of spores produced per colony (Spoc), with leaf age as a factor. For Diam and Spoc, some of the age classes were grouped to allow for statistical comparison when the sample numbers were too low (< 5) due to a failure of the infection process related to ontogenic resistance (only a few spores are deposited) (Table 2). The effect of leaf age on the independent variables was measured by performing Tukey-Kramer's multiple comparisons test adapted for unbalanced designs. The mean number of spores produced per area of a colony (Spoc/[(Diam/2)2.π]), a proxy of the sporulation capacity according to Lannou (2012), was compared by permutation testing (5000 permutations) for two classes of leaf age.

The disease data functions of leaf age were fitted to models using ordinary least squares methods. The choice of one specific model was based on Akaike's information criterion, which selects the model most likely to have generated the data. Goodness of fit was measured by *R*^2^ and the adequacy of the model by replicate testing. A fitting procedure was performed using Prism software (version 5.04).

Relationships among experimental conditions, leaf growth characteristics, leaf physiological indicators and disease were studied by a partial least squares path model analysis (PLS-PM) (Tenenhaus et al., 2005). The PLS-path model was described by six unobservable or latent variables (LVs): “Variety,” “Climate,” “Leaf growth,” “Leaf physiology,” “Inoculum,” and “Disease.” This analysis provides how much variation of “Disease” can be predicted by the other latent variables. Each LV was constructed by a set of observable variables called manifest variables (MVs). “Variety” and “Inoculum” were constructed in a formative way by qualitative variables (CS and M for variety, 2005–2009–2010 for inoculum), corresponding either to the varieties Cabernet Sauvignon and Merlot or to the year using different isolates. “Climate” was described in a reflexive way by the average temperature over the 10 days before the experimentation (STJ-10) and the sum of rain (SRJ-10). “Leaf growth” was described in a reflexive way by leaf age (LA), the sum of GD temperature during leaf development (LAdev) and the leaf surface (LS). It is worth noting that, for one experiment, leaves sharing the same leaf age encountered the same climatic conditions, but they do not necessarily have the same plastochron index, which is a function of the rate of growth of the shoot, nor do they have the same LS, which is dependent on the variety, vigor and environmental conditions. An identical leaf age yields various LAdev depending on experiments. “Leaf physiology” was described by the leaf soluble carbohydrates (SS), starch (ST) concentrations and LWC. The “Disease” variable was described by the variables characterizing the two basic pathogen processes: the infection efficiency (Inf) and the sporulation per colony (Spoc). The standardized latent variables were estimated as linear combinations of their centered MVs. Collinearity between manifest variables was allowed. The PLS-path model was described by the measurement model, which related the different MVs to their own LVs, and the structural model, which linked the endogenous LV “Disease” to the other LVs: “Leaf growth,” “Leaf physiology,” and “Year.” The inner estimate was performed using a path weighting scheme algorithm. Confidence intervals on regression coefficients are estimated by bootstrap methods (500 random sampling of 280 individuals of the observed data set). Two criteria of the goodness of fit of the model (GoF) are provided to evaluate the external model (relationship between MVs and LVs) and the internal model (relationship between LVs). Altogether, 321 observations (leaves) were considered (49 from 2005_exp1, 64 from 2005_exp2, 117 from 2009_exp4, 50 from 2010_exp5, and 41 from 2010_exp6). Observations on leaves older than 31 days were not considered to avoid introducing bias between the early and late experiments. Missing values were estimated using NIPALS procedures. The analysis was performed using the XLstat PLS-PM module (version 2012.2.02).

Finally, logistic regression was performed on the whole data set, including old leaves. The Spo variable was transformed into binary form with a value of 1 for Spo >3000 (significant peak with the particle counter) and 0 for less. Then, a model was fitted to describe how the chance of the event of a “diseased” leaf occurring depends on a maximum of one or two covariables, among which are “LA,” “LAdev,” “LS,” “SS,” “ST,” and “1/LWC.” The regression assumes that *P*(*Y* = 1/*X* = *exp*(β_0_ + β_1_*X*_1_ + β_2_*X*_2_)/*exp*(β_0_ + β_1_*X*_1_ + β_2_*X*_2_) with Y the outcome and X the covariates. The model parameters β are estimated by the method of maximum likelihood. The analysis was performed on 497 individuals from the 5 experiments [261 leaves classified as “healthy” = “non-sporulating”; and 236 classified as “diseased”; null hypothesis P(D) = 0.47]. Among these leaves, 30 were randomly sampled for model validation. The ability of the model to distinguish between low and high risk for a leaf to be diseased was tested by ROC (receiver operating curve) curve analysis. The ROC curve plots the sensitivity (proportion of truly “positive,” here “diseased,” observations classified as such by the model) function of the 1-specificity (the specificity is the probability of the model predicting the sample “negative,” given that the observation is “negative,” here “healthy”). The area under the curve yields the ability of the model to correctly distinguish between healthy and diseased leaves. When the AUC is high (>0.8), the model has high discrimination ability.

## Results

### Environmental Growing Conditions

**Figure 1:**
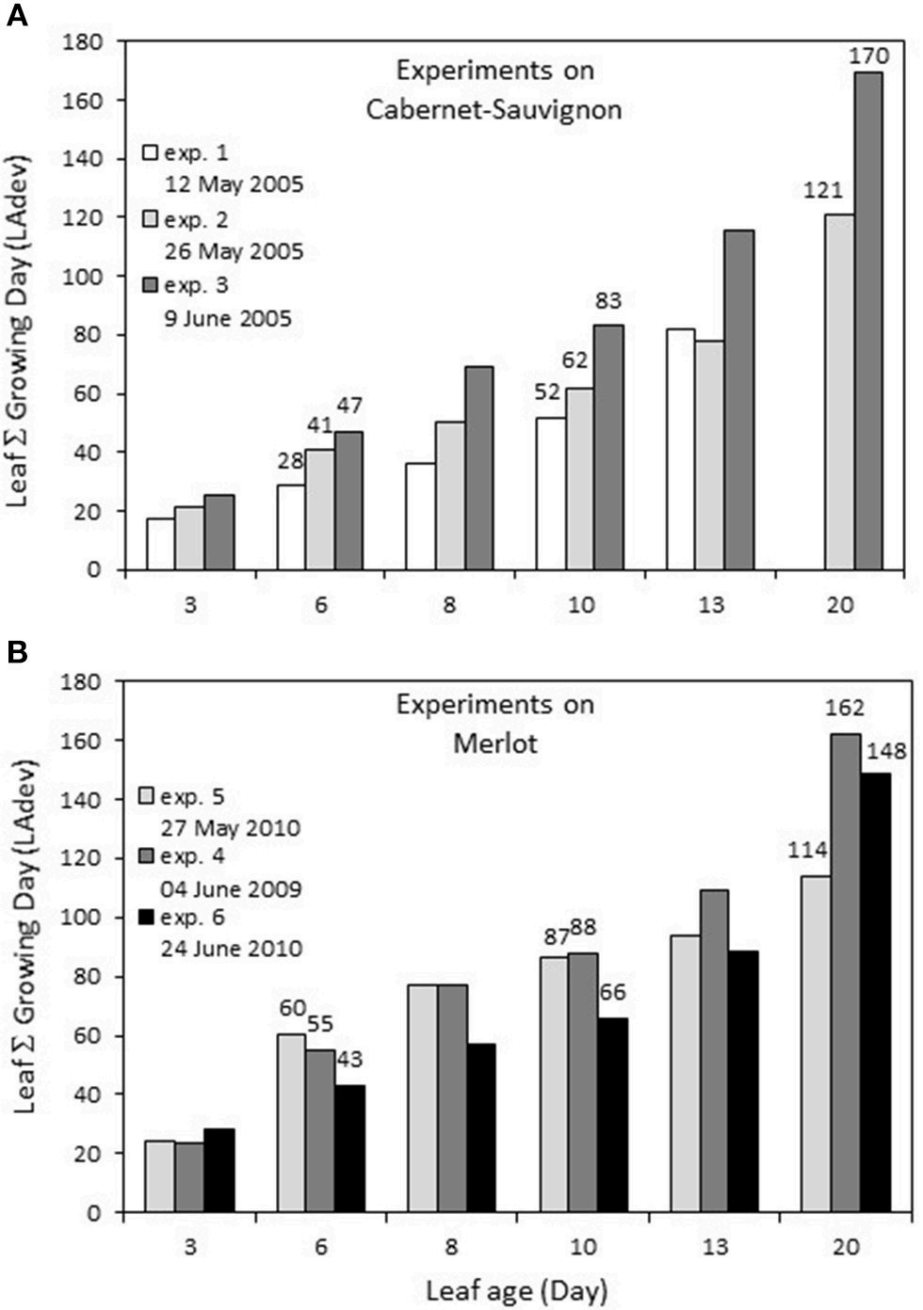
Sum of growing days (Sum of daily temperature >10°C) encountered by a leaf during its development depending on its age, for experiments on Cabernet Sauvignon **(A)** and Merlot **(B)**. Experiments on Cabernet-Sauvignon were conducted on 12, 26, May and 9 June 2005 (exp. 1, 2, 3) and experiments on Merlot were conducted on 4 June 2009, 27 May and 24 June 2010 (exp. 4, 5, 6).

The first leaves were marked on April 29 for Cabernet Sauvignon (2005, exp. 1–2–3), corresponding to a sum of 138.7°C growing degrees (GDs) and on April 14 and April 19 for Merlot, corresponding to 52.1°C GD (2009 exp. 4), and 66.2°C (2010 exp. 5–6), respectively. For each experiment, there was a linear relationship between leaf age (LA) and the sum of GD for a leaf during its development (LAdev), with an increased difference for older leaves. The GD globally explained the number of leaves per shoot for each experiment, with ~21°C for each newly expanded leaf. For Cabernet Sauvignon, the leaves sampled for experiment 1 (early May 2005) were those developed at the coldest temperature: a 13-day-old leaf developed under a similar GD as a 10-day-old leaf from experiment 2 (Figure 1). In comparison, for Merlot, the leaves sampled for exp. 6 (late June) encountered the coldest period: 13-day-old leaves developed under a similar GD as 10-day-old leaves from exp. 5 (end of May).

### Infection Rate Function of Leaf Age

**Figure 2:**
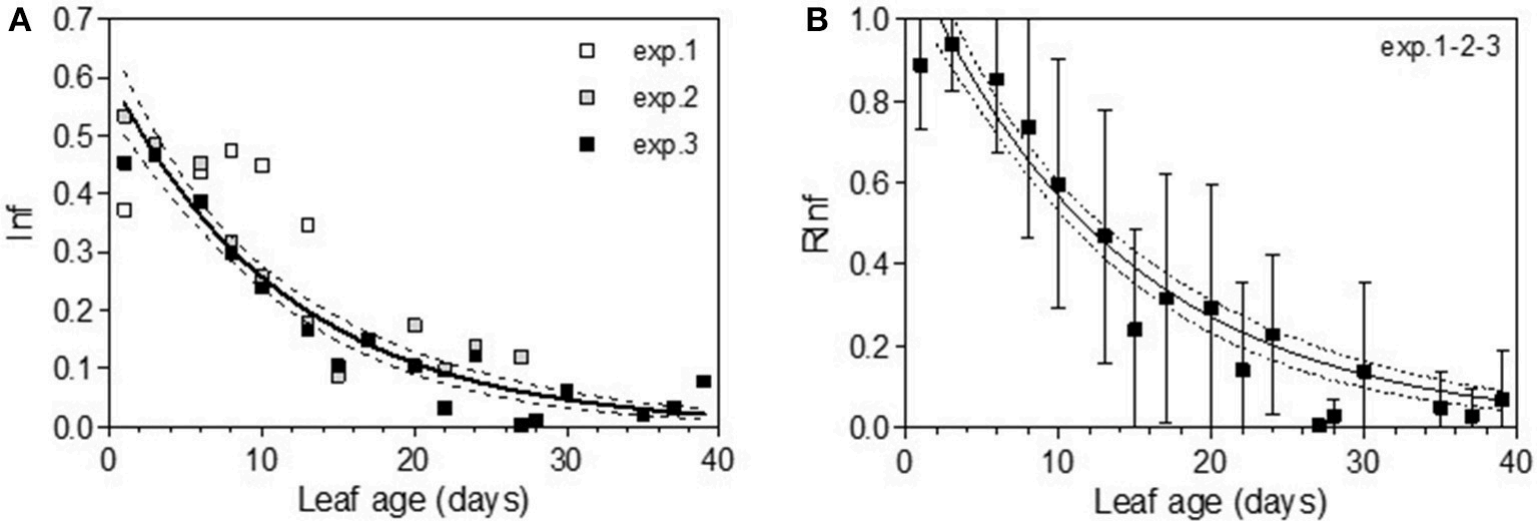
Infection efficiency rate (Inf) **(A)** and relative infection efficiency rate (RInf) **(B)** as a function of leaf age for experiments on Cabernet Sauvignon. Bars indicate standard deviations. Plain lines correspond to the fitted model M1 in Table 4. Dashed lines correspond to the 95% confidence bands on the models.

In 2005, for the first sampling date (exp. 1), no significant difference in infection efficiency was observed between the different leaf ages of Cabernet Sauvignon (Table 3), whereas for the second (exp. 2), and third experiments (exp. 3), which occurred later in the season, the youngest leaves up to 6 days old displayed significantly higher infection rates. For experiments 2 and 3, we could adjust the data of infectious efficiency to the same exponential decrease model (Figure 2A, Table 4-M1). In such a model, 10-day-old leaves had an infection efficiency that decreased to 0.25. When the data were standardized (data divided by the maximum % of infection on the shoot), the whole set of data could be adjusted to a decreasing exponential model with an *R*^2^ of 0.59 (Figure 2B, Table 4-M1). Thus, a 10-day-old leaf lost approximately half of its infectivity, compared to the most susceptible ones. For the Merlot cultivar, the rate of infection efficiency also showed an exponential decrease with leaf age. Very young leaves were excluded from the analysis since we could not exclude that the tape test was not disturbed on particularly hairy young leaves (Figure 5A, Table 5-M1). For experiment 4 (4 June 2009) and 5 (27 May 2010), with similar environmental conditions, the decrease with leaf age was quite similar, despite a higher rate of infection for very young leaves (I_0_) in experiment 4. For experiment 6 (24 June 2010), leaves up to 16 days old that developed at cooler temperatures showed a high rate of infection, and then the rate decreased very quickly.

**Table 3:** Effect of leaf age of Cabernet Sauvignon on the disease variables tested by variance analyses and mean comparisons for each leaf age level.

	**Exp.1**				**Exp.2**				**Exp.3**			
**Leaf age**	**Inf**	**Spo**	**Spoc**	**Diam**	**Inf**	**Spo**	**Spoc**	**Diam**	**Inf**	**Spo**	**Spoc**	**Diam**
1	0.373^a^	20569.8^ab^	8447.9^a^	6.31^ac^	0.534^a^	16675.5^b^	14935^a^	7.15^a^	0.442^a^	34328.6^a^	12920^a^	8.06^a^
3	0.476^a^	24806.9^ab^	_________	_________	0.467^a^	45861.6^a^	_________	_________	0.468^ab^	25283.1^a^	_________	_________
6	0.436^a^	32483.6^a^	8600.0^a^	5.90^ac^	0.434^ac^	53627.0^a^	12108^ac^	7.8^a^	0.387^ab^	19720.7^ab^	18280^ab^	7.39^ab^
8	0.474^a^	21340.9^b^	8431.9^a^	6.50^a^	0.292^ab^	13138.9^b^	6965^bc^	6.58^ac^	0.299^abc^	18105.5^abc^	12600^abc^	6.96^ab^
10	0.457^a^	7438.0^c^	4112.4^b^	5.34^bc^	0.214^b^	8294.5^b^	3673^b^	4.41^bc^	0.249^bc^	8992.7^bcd^	5655^c^	4.63^c^
13	0.349^a^	1974.5^c^	3915.6^b^	5.25^bc^	0.173^b^	7739.5^b^	4518^b^	4.88^bc^	0.170^bcd^	4010.196^d^	6664.3^c^	4.78^c^
15					0.090^b^	8752.4^b^			0.103^cd^	1227.4^d^	
17					0.149^b^	1864.4^b^			0.143^cd^	3362.370^d^		
20					0.238^bc^	5820.6^b^			0.105^cd^	3518.1^d^	
22					0.113^b^	8394.9^b^	_________	_________	0.033^d^	3324.0^d^	_________	_________
24					0.176^b^	4857.2^b^	3510 ^b^	4.62^bc^	0.121^cd^	4363.1^bcd^	7590^bc^	3.75^c^
27					0.134^b^	4857.2^b^			0.003^d^	_________	_________	_________
29–39									0.048^d^	4275.05^cd^	7850^bc^	5.75^bc^
df model, error	5, 43	5, 88	5, 37	4, 44	11, 95	11, 114	5, 43	6, 56	12, 144	11, 76	6, 16	6, 36
F fisher	1.71	27.54	8.41	4.49	10.73	48.24	16.93	8.72	13.35	8.37	11.03	14.1
*P*-value	0.154	<0.0001	<0.0001	0.0039	<0.0001	<0.0001	<0.0001	<0.0001	<0.0001	<0.0001	<0.0001	<0.0001

*Inf, percentage of infectious spores; Spo, sporulation of inoculated leaf disks; Spoc, sporulation of the colony; Diam, maximal colony diameter. Different letters near the average indicate significant difference at P <0.05 according to the Tukey-Kramer multiple comparison test*.

**Table 4:** Results of model fitting for disease variables for experiments on Cabernet Sauvignon.

**Model equation**	$Y=I_{0}{\text{exp}}^{-kX}$				**$Y=m+\frac{M-m}{1+10^{\left(X-q50\right)}}$**		$Y=B+A{\text{exp}}^{\left[-\left(\frac{\left(X-c\right)}{D}\right)\right]2}$						***Y*** = *a*exp^*bX*^
	**with Y = Dependent variable and X = leaf age variable**				**with Y = Dependent variable and X = leaf age variable**		**with Y = Dependent variable and X = leaf age variable**					**with Y = Dependent variable and X = leaf age variable**
Dependant variable		Inf	RInf		Diam		Spo	Spo	Spo	Spoc	RSpo		Spoc
Model identification		M1	M1		M2		M3	M3	M3	M3	M3		M4
Experimentation		exp.2-3	exp.1-2-3		exp.1-2-3		exp.1	exp.2	exp.3	exp.1-2	exp.1-2-3		exp.2
df		209	231		120		56	91	66	64	182		41
Parameter value	*I_0_*	0.606	1.181	*m*	4.64	*A*	34357	54676	32600	12673	0.95	*a*	515.5
	*k*	0.086	0.073	*M*	6.86	*B*	2124	7498	3628	3029	0.12	*b*	0.4
				*a50*	8.35	*c*	5.151	4.769	0.699	4.455	4.659	
						*D*	3.479	2.957	6.666	3.566	3.65	
Goodness of fit R^2^		0.57	0.59		0.41		0.76	0.85	0.79	0.72	0.88		0.74
*P*-value for Replicates tests		0.7	0.1		0.71		0.7	0.6489	0.96	0.68	0.26		0.81
Evidence of inadequate model		No	No		No		No	No	No	No	No		No
Outliers		0	0		0		4	1	6	0	0		0

*Inf, rate of infectious spores (branched conidia at 72hpi); RInf, relative rate of infectious spores; Diam, colony diameter (mm); Spo, number of spores generated per cm^2^ of inoculated leaf area; Spoc, number of spores generated per colony; RSpo, relative rate of spores generated per cm^2^ of inoculated leaf*.

**Table 5:** Results of model fitting for comparison of infection rate (Inf), colony diameter (Diam), and sporulation rate (Spo) for experiment on Merlot.

**Model equation**		$Y=I_{0}{\text{exp}}^{-kX}$				$Y=m+\frac{M-m}{1+10^{\left(X-q50\right)}}$				$Y=B+A{\text{exp}}^{\left[-\left(\frac{\left(X-c\right)}{D}\right)\right]2}$							***Y*** = *a*exp^*bX*^	
						**With** ***Y*** **= Dependent variable and** ***X*** **= leaf age**				**With** ***Y*** **= Dependent variable and** ***X*** **= leaf age**							**With** ***Y*** **= Dependent variable and** ***X*** **= colony diameter**	
**Dependant variable**		**Inf**	**Inf**	**Inf**		**Diam**	**Diam**	**Diam**		**Spo**	**Spo**	**Spo**	**Spoc**	**Spoc**	**Spoc**		**Spoc**	**Spoc**
Model identification		M1	M1	M1		M2	M2	M2		M3	M3	M3	M3	M3	M3		M4	M4
Experimentation		exp 4	exp 5	exp 6		exp 4	exp 5	exp 6		exp 4	exp 5	exp 6	exp 4	exp 5	exp 6		exp 4	exp 5-6
df		137	56	85		40	22	24		131	63	91	139	61	94		43	45
Parameter value	***I**_**0**_*	0.63	0.38	2243	***m***	4.91	5.15	2.67	***A***	73207	55842	62308	20134	2895	7240	***a***	883.7	241.3
	***k***	0.095	0.067	0.615	***M***	9.93	8.15	10.3	***B***	2606	2312	449.5	807.6	756.9	210.2	***b***	0.29	0.26
					***a50***	12.17	12.61	16.93	***c***	8.93	6.51	13.29	8.36	6.28	11.99			
									***D***	5.14	3.68	3.47	4.68	3.13	3.24			
Goodness of fit *R*^2^		0.74	0.54	0.75		0.66	0.24	0.69		0.81	0.72	0.89	0.6	0.21	0.67		0.54	0.5
*P*-value for Replicates		0.55	0.11	0.79		0.35	0.45	0.72		0.37	0.98	0.34	0.99	1	0.99		0.89	0.21
Evidence of inadequate model		No	No	No		No	No	No		No	No	No	No	No	No		No	No

### Colony Growth Function of Leaf Age

**Figure 3:**
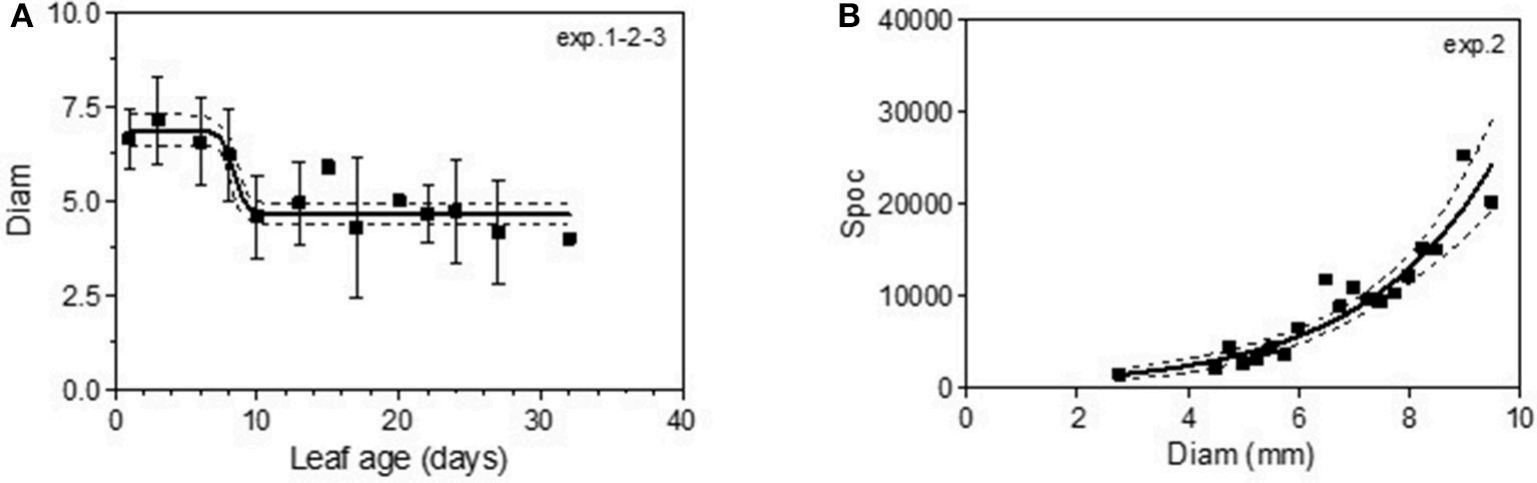
Colony diameter in mm (Diam) as a function of leaf age after 14 days of colony growth **(A)** and number of spores produced per colony (Spoc) as a function of the colony diameter **(B)** for Cabernet-Sauvignon. **(A)** correspond to the average colony diameter from experiments 1–3 whereas Panel **(B)** to the spores per colony from experiment 2. Plain lines correspond to the fitted models M2 **(A)** and M4 **(B)** in Table 4. Dashed lines correspond to the 95% confidence bands on the models, and bars correspond to the standard deviation.

The maximum diameter of a colony produced by single conidia was, on average, smaller on leaves older than 10 days for Cabernet Sauvignon (Table 3). A model with a decreased diameter at the inflection point of 8.35 days (a_50_), a maximum diameter (M) on young leaves of 6.8 mm and a minimum diameter (m) of 4.6 mm for older leaves, fit the data with an *R*^2^ = 0.41 (Table 4-M2, Figure 3A). On average, the sporulation of a colony was exponentially correlated with the maximal diameter attained by the mycelium network (Table 4-M4, Figure 3B), but at < 5 mm, the level of sporulation was low and constant, indicating that a colony with only a two-millimeter difference in diameter (e.g., between 5 and 7 mm) will generate twice as many more spores. For Merlot, the trends and models were the same but with different parameters depending on the experimentation. In experiments 4 and 6, the maximum diameters were greater than in experiment 5. Similar to infection efficiency, slower growth appeared for older leaves in experiment 6 (a50 = 16.93 vs. 12.6 or 12.17 for exp. 5 and 4) (Table 5-M4, Figure 5B). Sporulation per colony was much higher for experiment 4 (Figure 5D).

### Sporulation Function of Leaf Age

**Figure 4:**
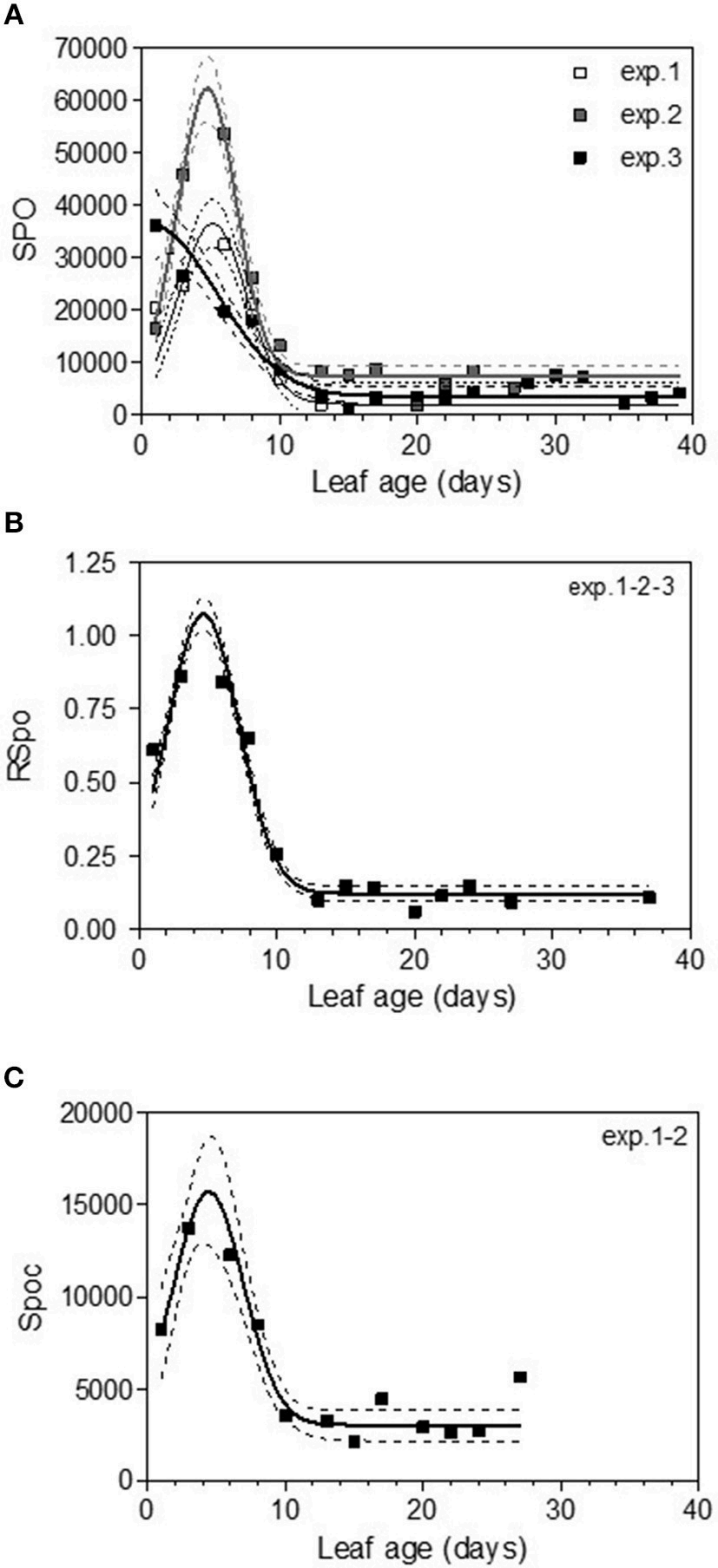
Sporulation (number of spores produced per cm2 of infected leaf disk) (Spo) **(A)**, relative sporulation (RSpo) **(B)** and number of spores generated per colony (Spoc) **(C)** as a function of leaf age for experiments on Cabernet Sauvignon. Plain lines correspond to the fitted model M3 in Table 4. Dashed lines correspond to the 95% confidence bands on the models, and bars indicate standard deviation.

**Figure 5:**
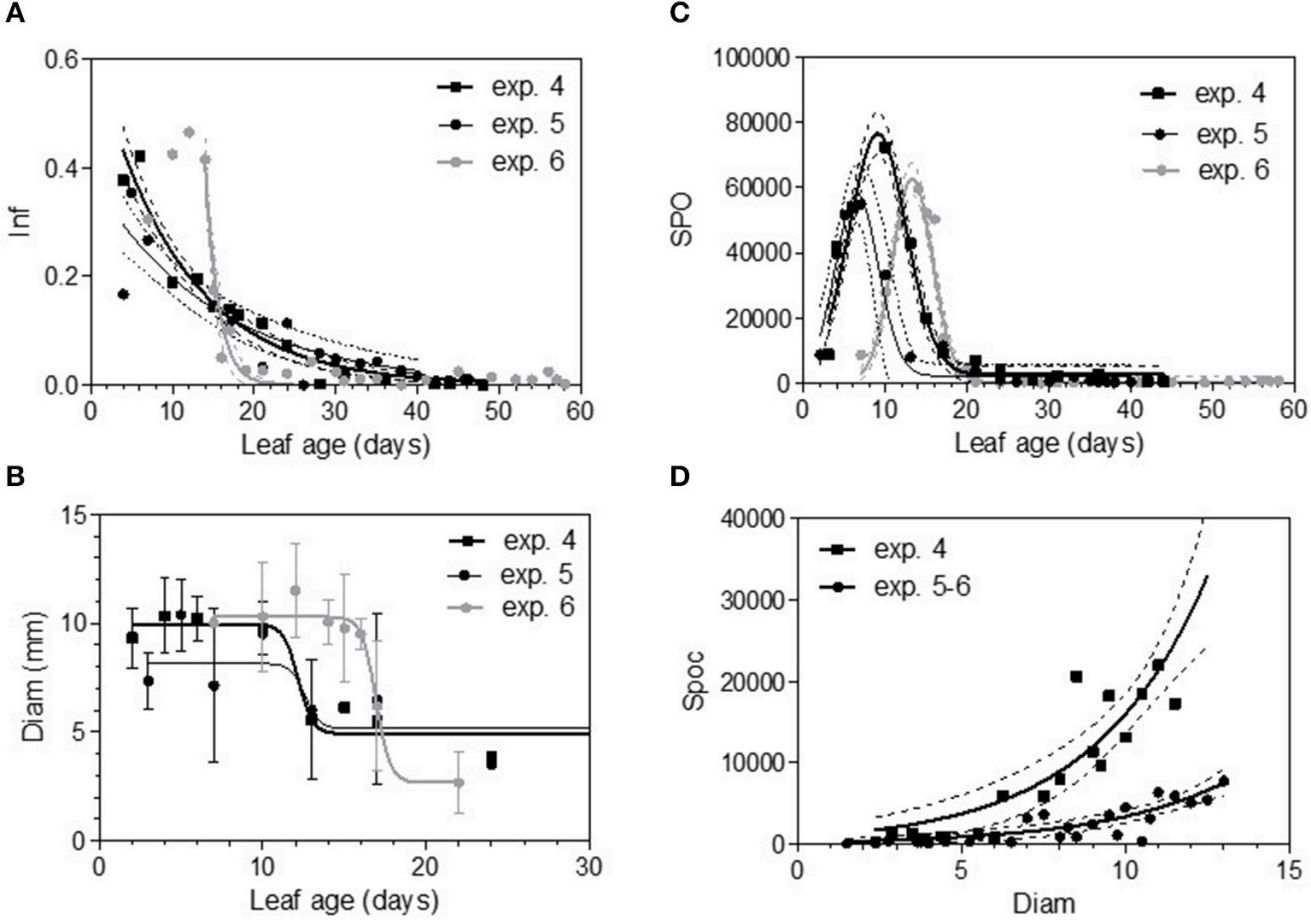
Infection efficiency rate (Inf) **(A)**, colony diameter (Diam) **(B)**, and sporulation (number of spores produced per cm2 of infected leaf disc) (Spo) **(C)** as a function of leaf age and sporulation per colony (Spoc) as function of colony diameter **(D)** for Merlot experiments. Plain lines correspond to the fitted models M1 **(A)**, M2 **(B)**, M3 **(C)**, and M4 **(D)** in Table 5. Dashed lines correspond to the 95% confidence bands on the models, and bars correspond to the standard deviation.

The variable sporulation (Spo) measured on inoculated leaf discs results from three successive processes: infection efficiency, mycelium growth and sporulation. From this point of view, it could be considered an integrative variable of susceptibility. The Spo variable followed a clearly bell-shaped curve function of leaf age for experiments 1 and 2, with higher levels of spores produced on 3- to 6-day-old leaves and a decrease for 8-day-old leaves (Figure 4A, Tables 3, 4-M3). For experiment 3, on average, the youngest leaves had the highest level of sporulation. When the three experiments were pooled and standardized to homogenize the shoots, the same bell-shaped curve was obtained (Figure 4B, Table 4-M3) with a high level of sporulation for leaves aged between 3 and 8 days, corresponding to a plastochron index (LPI) between 1.3 and 1.7 (first to second leaf expended). Sporulation per colony (Spoc), which is independent of infection efficiency, also had this bell-shaped curve (Figure 4C, Table 4-M3). For experiment 2, for which the data were most numerous, the number of spores produced per area of sporulating colony (the sporulation capacity) was significantly higher (P = 1) for very young, susceptible leaves (1–6 days), compared to older leaves (≥10 days) (295 sp.mm2 against 167 sp.mm2). This finding is consistent with the hypothesis that, for very young leaves, the higher sporulation results from an increase rate in the hypha branching combined with an increased number of spores produced per conidiophore. For Merlot, the sporulation curve with leaf age was similar to that observed on Cabernet Sauvignon, but the stage of high sporulation (Spo and Spoc) lasted longer, with increased sporulation for 5- to 16-day-old leaves with a peak at 6, 9, or 14 days, depending on the experiment (Figure 5C, Table 5-M3). This outcome matched an LPI of 2.5 (exp. 6) to 3.3 (exp. 4).

### Leaf Physiological Evolution

**Figure 6:**
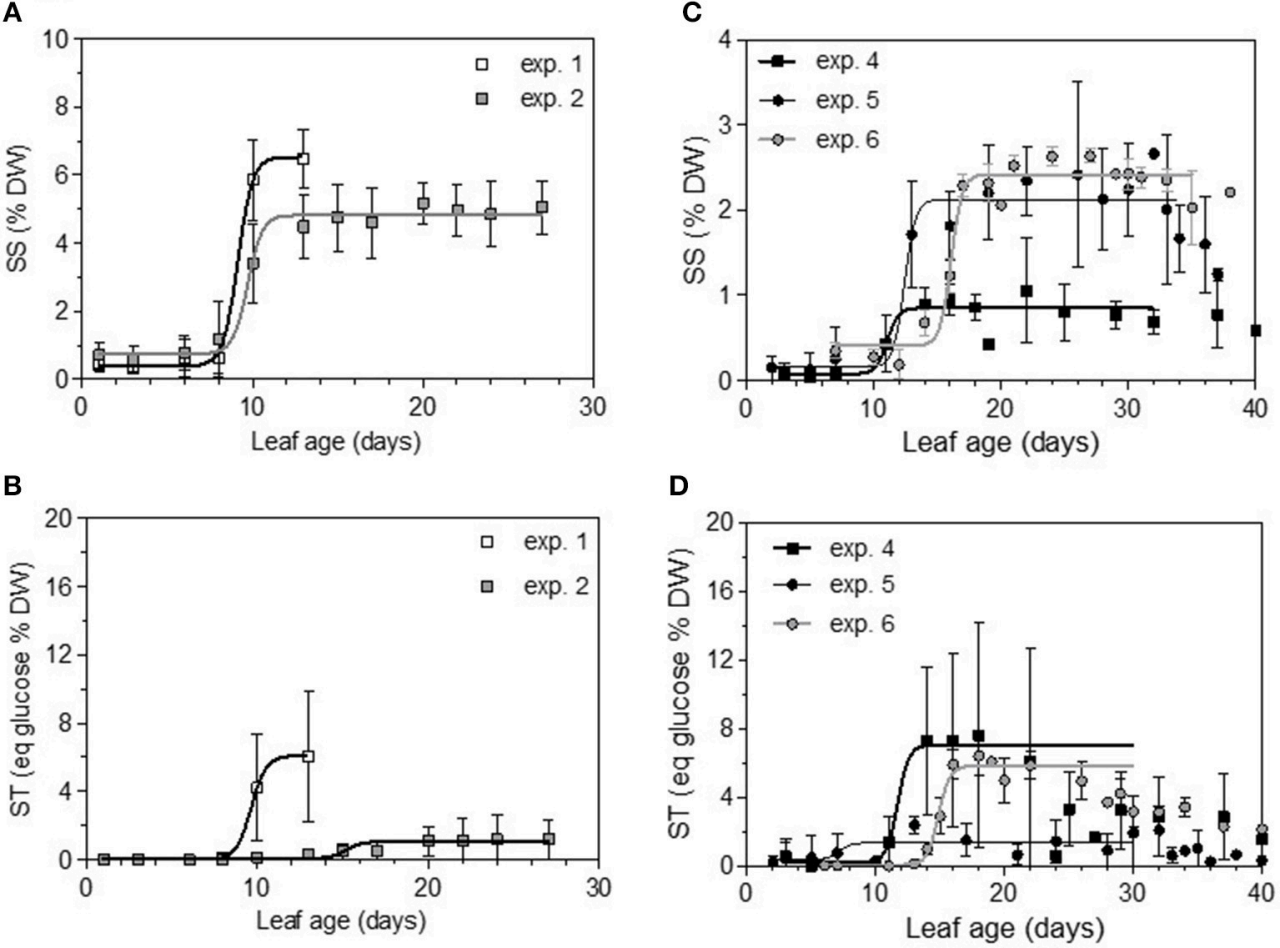
Soluble sugars (SS) and complex sugar (ST) concentrations expressed as percentages of leaf disc dry weight and as a function of leaf age for the two first dates of sampling on Cabernet Sauvignon **(A,B)** and Merlot **(C,D)**. Bars indicate standard deviations.

**Figure 7:**
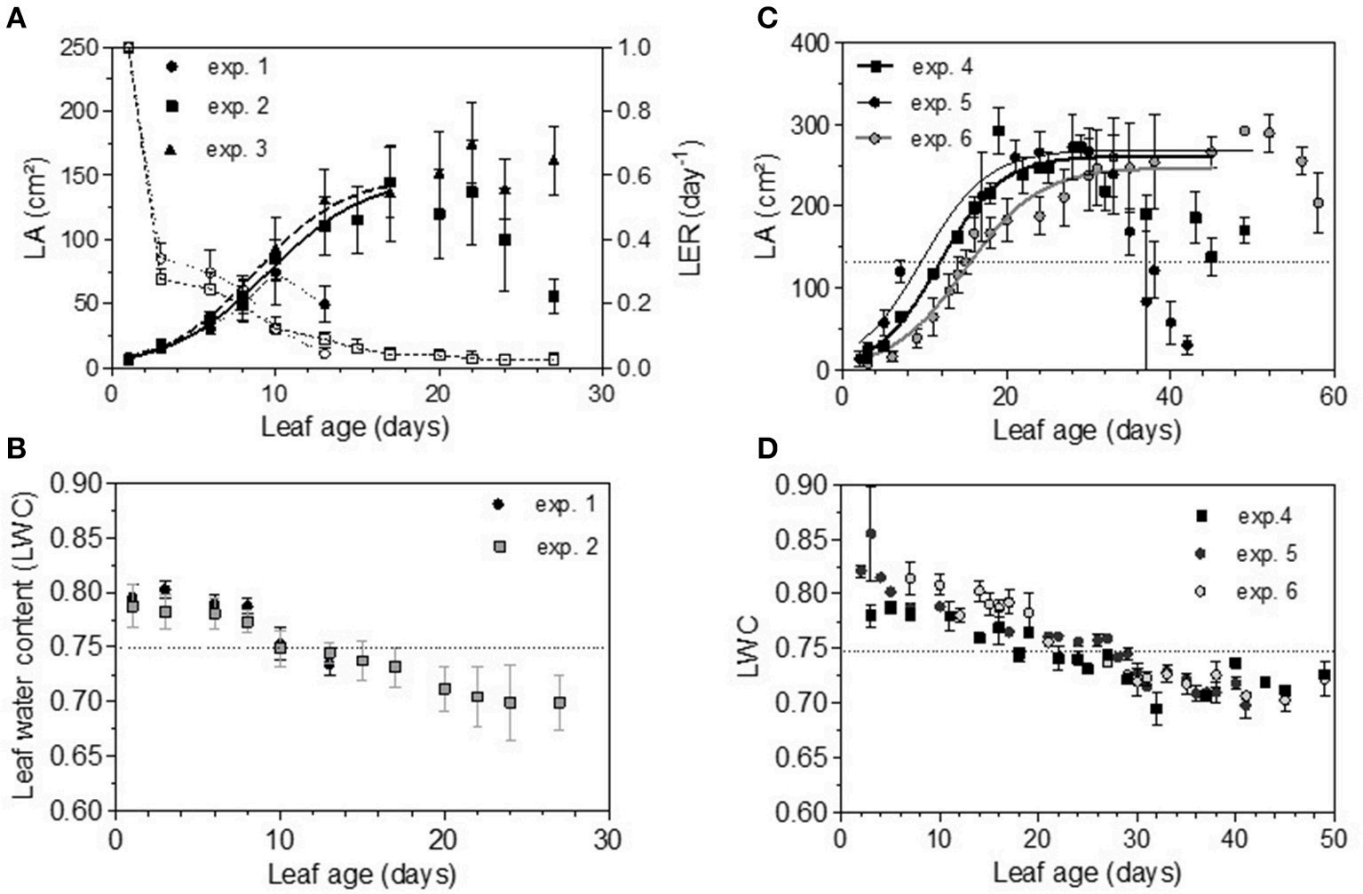
Evolution of leaf area (LA), leaf extension rate (LER) (dashed lines) and leaf water content (LWC) for Cabernet Sauvignon **(A,B)** and for Merlot **(C,D)**. Bars indicate standard deviations.

Total non-structural carbohydrate (TNC) concentrations tended to follow sigmoid curves regarding leaf age. The soluble sugars concentration (SS) displayed a strong and rapid increase for young leaves, followed by a plateau for older ones. For Cabernet Sauvignon, this change appeared on 8- to 12-day-old leaves (LPI = 2.7 to 3.4), while for Merlot, it appeared later: 10- to 14-day-old leaves (exp. 4 and 5, LPI = 3.9) to 14-to 16-day-old leaves (exp. 6, LPI = 3.7) (Figures 6A,C). The level reached for old leaves depended on the experiments. The leaf starch concentration was also highly variable with leaf age, but no starch was detected within the youngest leaves (< 10 days) (Figures 6B,D). Leaves showing an increase in SS reached ~50% of their final size (~150 and 270 cm2 for, respectively, CS and M), consistent with the time for transition between sink-to-source tissue (Figures 7A,C). The increases in the leaf soluble sugar content and leaf age were associated with a decrease in leaf water content (Figures 7B,D).

Thus, the processes of infection efficiency, sporulation and colony growth associated with leaf age showed similarities between the experiments on Cabernet Sauvignon and Merlot: a lower rate of mycelium growth and the end of the peak of a high level of sporulation matching an increase of sugar (SS); in comparison, the decrease in infection efficiency was more gradual with leaf age, as was the decrease in leaf water content. Infection efficiency is more closely correlated with leaf age (−0.68), leaf water content (−0.41), and starch (−0.32) than is sporulation (−0.54, −0.32, −0.30). Sporulation per colony was also significantly correlated with soluble sugar (−0.25), although it was more influenced by the experiments on Cabernet Sauvignon, for which we had more colonies for a wider range of leaf ages. For Cabernet Sauvignon, the proxy of the sporulation capacity (number of spores produced per unit area of sporulating colony) was significantly correlated with leaf age (−0.52) and with the amount of sugar (−0.64), indicating that colonies on young leaves with smaller amounts of sugar are significantly able to produce more spores per colony. When a period of cold is encountered, processes are delayed (increased sugar, leaf development, decreased leaf water content), and the duration of the sensitivity period is lengthened (exp. 6). Very early in the growing season (exp. 1), all of the leaves are as susceptible as each other to infection.

### Disease Severity Prediction

**Figure 8:**
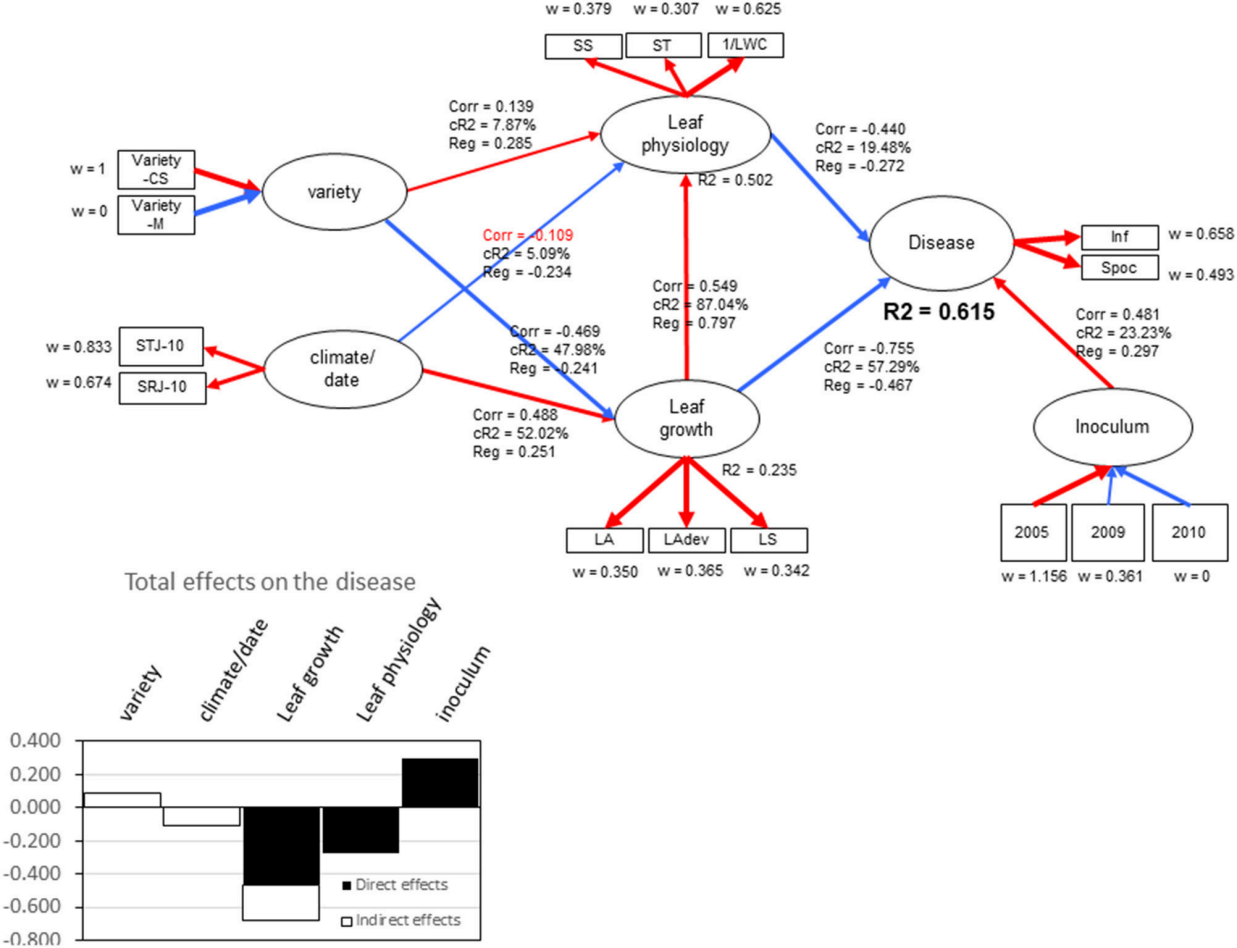
Output of partial least squares path modeling for the two years of experiments describing the relationships between the endogenous latent variable “disease” to the other latent exogenous variables “Leaf growth,” “Leaf Physiology,” “Inoculum,” “Variety,” and “Climate/date.” Corr indicates the correlation coefficient between two latent variables, CR2 indicates the relative contribution of exogenous latent variables to endogenous one, Reg indicates the regression coefficient, *R*^2^ indicates the regression coefficient between two latent variables, and w indicates the outer weights yielding the covariances between the manifest variable and its inner latent variable. Blue lines indicate negative correlations, and red lines indicate positive correlations.

Partial least-squares path modeling allowed for highlighting the relative contributions of the studied composite variables (“Leaf growth” and “Leaf physiology”) in the global explanation of the global “Disease” variable, depending on the experimental conditions (“Variety,” “Climate,” “Year”). The “Disease” variable was explained at *R*^2^ = 0.61 by “Leaf growth,” “Leaf physiology,” and “Year,” with relative contributions of 57.3, 19.5, and 23.2%, respectively (Figure 8). Variations in leaf physiology were explained at *R*^2^ = 0.50, mainly by leaf growth (87%) (leaf age, leaf temperature for development and leaf surface). “Variety” and “Climate” only poorly contributed to leaf physiology level. When considering total effects (indirect plus direct effects) on the disease, “Leaf growth” had a higher effect on “Disease” (−0.68) compared with “Leaf Physiology” (−0.27), due to the correlation between variables. Leaf water content (1/LWC) and simple sugars (SS) made the greatest contributions to “Leaf physiology” (w = 0.62 and 0.38, respectively), indicating that, regardless of whether sugar (SS) increases or water content decreases, the “Disease” variable decreases. The “Year” effect certainly reflects the variation of isolate aggressiveness that was obvious when considering the level of sporulation per colony diameter. The relative goodness of fit of the model was high (GoF_rel_ = 0.85) with moderate absolute goodness of fit (GoF_abs_ = 0.55), indicating that the predictors used only partly explained disease variation.

Logistic regression showed that, with a maximum of one co-variable, leaf age was the best predictor (Pr > LR < 0.0001, β_0_ = 4.12, β_1_ = −0.20), with sensitivity of 86.02% (73.3% for the validation sample) and very high discrimination (AUC = 0.927) of “diseased” vs. “not diseased” leaves. The individuals not well assigned were those at the transition state or very young leaves that were not sufficiently sporulating but that were considered susceptible by the model. If we analyzed the probability after model fitting, there was a probability for a leaf to be susceptible of 0.8 for an average leaf age of 13.3 days [11.4; 14.9] and a probability decrease at 0.5 for a leaf age of 20 days [18.7; 21.4]. With two co-variables, leaf age and starch content were the best predictors, but the model was only slightly improved (AUC = 0.936) with slightly higher specificity (prediction that the leaf is healthy given that it is observed to be healthy).

## Discussion

Understanding and quantifying the process of ontogenic resistance of the grapevine leaf of the two susceptible, first widely cultivated wine cultivars Cabernet Sauvignon and Merlot to one of the major fungal pathogens, *E. necator* is a prerequisite to promote more sustainable pest management strategies in viticulture. Although ontogenic resistance on grapevine bunches has been well documented (Ficke et al., 2002; Gee et al., 2008), little evidence is available of similar processes for leaves, although leaves are the primary targets for the onset of epidemics. In this study, we attempted to analyse and quantify how the life traits of the pathogen were impacted by ontogenetic resistance and find predictors on the plant side associated with them. To obtain a generic view of the phenomenon, we used shoots coming from vinestocks grown in vineyards under different environmental conditions, and we used several predictable variables that were more or less easy to measure and relevant to analyse leaf state change.

Pathogen behavior was analyzed in relation to leaf age and was characterized by: (i) highly infectious colony growth and sporulation rates for young leaves (< 10 days for Cabernet Sauvignon and < 16 days for Merlot), with significantly higher sporulation on very young leaves; and (ii) lower colony growth, with a strong decrease in the sporulation rate and in the relative infection rate for older leaves (>10–16 days). Then, all of the chronologic infectious steps of the pathogen—infection efficiency, mycelium growth and sporulation—are affected by the leaf age. The sporulation capacity is even increased on very young leaves, at least for Cabernet Sauvignon. This increase in sporulation was correlated to low sugar content, whereas the decrease in infection efficiency was more closely correlated with a decrease in leaf water content and an increase of starch. If we consider the leaf plastochron index, instead of leaf age, compared with other studies, the peak of sporulation was 1.4 for Cabernet Sauvignon and 2.7 for Merlot. These values of LPI varied by less than one point for the average temperature encountered during the experiments (15.9–18.7°C), indicating that, at each time during the growing season, very few leaves are potentially highly susceptible (~2 to 3 per shoot), and the landscape of susceptibility should be higher for Merlot than for Cabernet Sauvignon. In a previous study (Merry et al., 2013), Cabernet Sauvignon leaves showing the maximum severity were those at LPI 3.7 at 18°C (constant temperature in the glasshouse), and the range of maximum severity was for leaf positions 2.1- 4.8. At 25°C, for a higher rate of leaf emergence, the mean modal leaf position for maximum severity was 4.4. Comparatively, in our study with leaves coming from field shoots under natural irradiance and fluctuating temperatures, the maximum severity (corresponding more or less to Spo) was earlier (1.4), with the decrease of leaf susceptibility even sharper. In another study performed on cuttings in growth chambers, colony hyphal length and infection efficiency decreased on both resistant and susceptible cultivars as leaves matured (LPI 1 to LPI 8), with, however, high variations between cultivars (Doster and Schnathorst, 1985).

The high infection rate and increased colony growth on young leaves resulted in an increase in penetration sites and haustoria formation favoring pathogen nutrient uptake and, consequently, a higher sporulation rate. In our study, the very young, susceptible leaves were characterized by a small amount of glucose content, higher water content and a high leaf extension rate. These expanding leaves reached only ~1/3 of their final size, consistent with the status of sink tissues for grapevines (Lebon et al., 2008; Scott, 2008). Sink leaves depend on other leaves for their energy supply, and the carbohydrates that they receive from source plant parts are immediately used for respiration, growth and the synthesis of new cell components and are therefore not stored. Sink leaves correspond to leaf plastochron indices of 1–3, showing maximal photosynthetic activity (30% for LPI 3) (Zufferey et al., 2000) and higher day respiratory rates (Rd) over the whole growing period. Rd values in young leaves (LPI 3-5) on primary shoots were two times greater than those of mature leaves (Zufferey, 2016). The leaves in transition from sink to source showed a high increase in sugar levels and were able to export sugars when the supply in carbohydrate exceeded the needs for metabolism. In our study, this transition matched decreases in mycelium growth, sporulation and infection efficiency when the number of source leaves exceeded that of sink leaves. When it was not the case, early in the growing season (like for experiment 1), the infection efficiency was not affected by leaf age, and only sporulation decreased for the two basal leaves. The correspondence between the sink to source transition and susceptibility to powdery mildew were once identified on leaves from Cabernet Sauvignon cuttings by autoradiography after ^14^CO_2_ labeling (Merry et al., 2013). Our study provided some clues about the ontogenic resistance process by decomposing the sequential development of the pathogen (infection rate, colony growth and sporulation), which converges toward a specific age-related transition in the receptivity of the leaf tissues. The complete life cycle of the fungus (up to sporulation) is restricted to sink tissue (small amount of sugar), while the first steps (infection, colony growth) can extend later. Based on these experiments for different environmental conditions and two varieties, we can accurately predict the susceptibility of the leaf with its age as a predictor: a leaf aged on average 13.3 days had a very high probability (0.8) of being susceptible, while this probability dropped to 0.5 one week later. Decreases in incidence and severity of powdery mildew with leaf age or development stage were also identified on strawberry (Carisse and Bouchard, 2010) and were reported to be a robust and biologically consistent process with a response consistent across cultivars and isolates employed and between the various sites tested (Asalf et al., 2014). Regarding pea powdery mildew, a resistance gene involved in post-penetration cell death and a reduction in percentage penetration success in mature leaves confers complete resistance in the field under high temperatures or on mature leaves (Fondevilla et al., 2006). On *Eucalyptus grandis*, all of the steps of the development of the rust *Puccinia psidii*—germination, appressorium formation, penetration and sporulation—were altered with increased leaf age (Xavier et al., 2015). For other pathosystems, the susceptibility can increase with leaf age, as is the case for another rust, *Melampsora larici populina*, which is more aggressive on mature leaves of poplar (Maupetit et al., 2018). The authors observed an increase in uredia (colony) size and a higher sporulation rate with increased LPI and lower sporulation capacity. In this case, pathogen traits were not correlated with leaf sugar, but there was a negative correlation with phenolic content, which was higher in younger leaves. On pea, detached stipules were more receptive *to Mycosphaerella pinodes* as soon as visual senescence was observed (Richard et al., 2012).

### Which Factors Are Involved in the Variation of the Ontogenic Resistance Process?

Our results attest to the repeatability of ontogenic resistance on leaves during vegetative growth for two susceptible cultivars. However, our results only explained 61% of disease variation. These variations can be linked to sampling shoots with differences in the implementation age of the ontogenic resistance process or between years/cultivars with variations in disease level. To explain variation between years, one parsimonious hypothesis is that it is based on differences in isolate aggressiveness. Indeed, a strong difference in sporulation for the same colony diameter was observed for different years. Variations between shoots and experiments depend on factors with effects on the sink-to-source status of the leaf, such as exposure to light before field sampling, crop load or even leaf damage. The transition to leaf resistance is, however, clearly related to the timing of the sink-to-source status rather than to the final level of glucose attained. Variations were also observed when leaves encountered a cold temperature, which slowed the physiological processes and delayed the timing of ontogenic resistance establishment (such as in experiment 6). We also observed that infection efficiency was not significantly different for the first leaves that emerged early in the season, which could emphasize the particular status of these first expanding leaves for which the source of assimilates used for growth first comes from retranslocation from the parent vine (Keller, 2015). Using ^14^C tracer, it has been shown that, at the 5-leaf stage shoot, only the first basal leaf is able to export assimilates to the youngest one, and at the 6-leaf stage, two to three basal leaves behave as sources (Yang and Hori, 1980). The experiments were conducted on a hybrid of Labrusca, but this outcome was consistent with what we observed. Indeed in our first experiment on Cabernet Sauvignon, only the two basal leaves being in the transition from sink to source showing significantly less sporulation. This should enhance infection success when epidemics start very early in the season and surely contributes to their particularly damaging effects.

Although infection efficiency is very low on old leaves, it is not always null. We do not know the impact of these rare infections considering that they were not sporulating at the time of assessment. In the pathosystems of apple/apple scab, diffuse colonies and mycelium that formed on old leaves continued to grow (Li and Xu, 2002), and these authors wondered whether these diffuse colonies could sporulate later in the season and become a source of mycelium for the formation of pseudothecia. On grapevines, we did not exclude that the translocation of carbohydrates from old leaves to berries during grape ripening would not modify leaf susceptibility or allow rare infected spores to continue their development and affect late colonies. This phenomenon has already been observed for other pathosystems, as especially demonstrated in the case of hermaphrodite plants, such as papaya (*Carica papaya* L.). Female fruit-bearing plants are susceptible to *Oidium caricae* on old leaves and fruit, whereas male plants are not attacked (Jarvis et al., 2002). These authors have advanced hypotheses of the shunting of assimilates from leaf to fruit to explain the differences. However, we could not observe any modification of ontogenic resistance processes after bunch closure (late July) on Merlot.

### What Hypotheses Can Be Advanced to Explain the Process of Ontogenic Resistance Observed on Old Leaves?

#### Role of Sugar

The increase in carbohydrate concentration in old leaves could be involved in the activation of secondary metabolism. Soluble carbohydrates are known to control the expression of various metabolic and plant defense-related genes (Koch, 1996; Rolland et al., 2006), and high sugar content can also suppress fungus degradation enzymes, such as grape chitinase (Saito et al., 2011). Powdery-mildew diseases have already been classified as “high-sugar resistance” pathogens (Vanderplank, 1984), indicating reversal of susceptibility to high levels of sugars. The relationship between sugar amounts and osmotic pressure might also be involved (Koroleva et al., 2002). Increased osmotic pressure can challenge the pathogen during the first step of infection, slowing tissue colonization by limiting the process of penetration of secondary haustoria. This scenario is consistent with our observed reduction of colony diameter on old leaves. Increased osmotic pressure was hypothesized to explain the greater resistance of mature leaf epidermal cells to other powdery mildews on apple (Cimanowski and Millikan, 1970) and peach (Schnathorst and Weinhold, 1957; Weinhold and English, 1964).

#### Activation of the PTI

Transcriptome and proteome analyses showed that the susceptible variety Cabernet Sauvignon is able to initiate basal defense mechanisms (Fung et al., 2008; Fekete et al., 2009; Marsh et al., 2010). Transcriptome analyses of leaves (Dufour et al., 2016) also revealed overexpression of defense genes (phenylalanine ammonialyase, stilbene synthase, anthocyanidin synthase, chitinase, lipase) on Cabernet Sauvignon leaves at ages of 15 and 20 days, compare to leaves at ages of 7 and 10 days, only after powdery mildew inoculation (unpublished data). For young expanding leaves, a high rate of cellular reactions of plant defense might be energetic and, for the carbohydrates, its consumption too costly (Bethany et al., 2014). MLO genes, known as “powdery mildew susceptibility genes,” might be involved in this regulation. It was hypothesized that adapted powdery mildew species release effectors that, by combining with specific MLO proteins, could suppress the host PTI (plant triggered immunity) in the young leaves (Dry et al., 2010). One hypothesis explaining ontogenetic resistance is that changes occur in the old leaves in the expression or activity of the MLO genes with the consequence of restoring the PTI (Qiu et al., 2015).

#### Constitutive Defenses

The transition in the trophic statute of the leaf bound up with development can also trigger the establishment of constitutive defenses to infection: cuticle thickness in *Lactuca sativa*, (Schnathorst, 1959) and *Prunus persica* (Weinhold and English, 1964); or the synthesis of antimicrobial compounds (Tattersall et al., 1997; Salzman et al., 1998). Precocious events of infection progress, such as germ tube initiation and appressorium differentiation, require physical or chemical cues (Muller and Riederer, 2005). Variations in cuticular waxes (quality and amount) during ontogenic development could be an inhibiting factor, consistent with the lower rate of conidium germination on old leaves that we observed. Constitutive defenses and PTI can be both active in source leaves.

### How Can Ontogenic Resistance Be Used in Epidemic Management?

Given the repeatability of the results, it is unlikely that the susceptibility of young leaves can be reduced without significant cost for the plant. Our results clearly demonstrated that powdery mildew takes advantage of the compromise that the plant must make between growth and defense (Bethany et al., 2014). Even if it is tempting to think that new genome editing techniques will allow for the easy knockout of genes, such as MLO, the effect of these knockouts on the physiology of the plant remain unknown (Acevedo-Garcia et al., 2014; Malnoy et al., 2016). The rate of both primary and secondary leaf appearance can, however, be slowed by the winegrower, hence reducing the proportion of available susceptible young tissues per unit of time. Indeed, agronomical practices, such as cover-cropping and the use of a vigor-controlling rootstock, can decrease the rate of leaf appearance and consequently disease level (Calonnec et al., 2009; Schnee et al., 2011; Valdes-Gomez et al., 2011). Simulations have shown that a reduction in grapevine vigor, through a decrease in both the numbers and the rates of secondary leaf appearance, can slow epidemics (Burie et al., 2011; Mammeri et al., 2014). Other practices, such as thinning of old leaves, will decrease the photosynthesis of younger leaves, thereby potentially delaying their ontogenic resistance, whereas shoot topping of younger leaves does not impact leaf photosynthesis per unit of leaf area (Petrie et al., 2003). Consequently, thinning might be less favorable to increasing leaf resistance than shoot topping, without considering the potentially positive effect of light on tissue receptivity (Zahavi and Reuveni, 2012). Other cultural practices, such as minimal pruning, which results in increased shoot numbers, reduce leaf and shoot size, and more synchronous leaves emergence (Poni et al., 2000), could allow the plant to escape disease by simultaneously causing all leaves to behave as a source. Such cultural practices could also be used to enhance the resistance of varieties with specific resistance genes. It would be interesting to revisit the theory of grapevine balance using tools of genetics and physiology in a general context of dieback and climate change, in which yields are sought, while maintaining control of pathogens that are very deleterious, such as mildews, is a prerequisite. Coupling functional structural grapevine physiology (Weinhold and English, 1964) and pathogen development models (Calonnec et al., 2008) would help to better understand crop susceptibility under various environmental conditions and complex canopies, while early protection of the foliage, which allows for desynchronizing of the disease cycle and the crop cycle toward a higher percentage of resistant leaves, should be preferred.

## Conclusions

Our results revealed a clear correlation between the appearance of grapevine leaf ontogenic resistance and the leaf transition state from sink to source. The soluble and complex sugars, leaf water content and leaves' extension rate constitute indicators of a change in the metabolic processes and are indirect markers of tissue age-related resistance. From a practical point of view, the additional effects of ontogenic resistance modification through crop management and resistant cultivars should be explored to obtain better control of powdery mildew epidemics in sustainable agriculture (Calonnec et al., 2013a). Moreover, the integration of this developmental process into a grapevine disease management program requires reconsideration of fungicide spraying with regard to the formatting of organ susceptibility windows.

## Author Contributions

AC: head of the research programme, data analyses, writer. JJ: experimental technician. PV: collaborator for physiology data analysis and experimentation. SS: post-doctoral student, experimenter, writer.

### Conflict of Interest Statement

The authors declare that the research was conducted in the absence of any commercial or financial relationships that could be construed as a potential conflict of interest.

## Supplementary Material

Supplementary Figure S1 Scheme of the experimental design.
