# Supplementary figures and images for: Pathogenicity Traits Correlate With the Susceptible Vitis vinifera Leaf Physiology Transition in the Biotroph Fungus Erysiphe necator: An Adaptation to Plant Ontogenic Resistance
Source: Front Plant Sci. 2018 Dec 11;9:1808. doi: 10.3389/fpls.2018.01808 (PMC6297386; doi:10.3389/fpls.2018.01808)

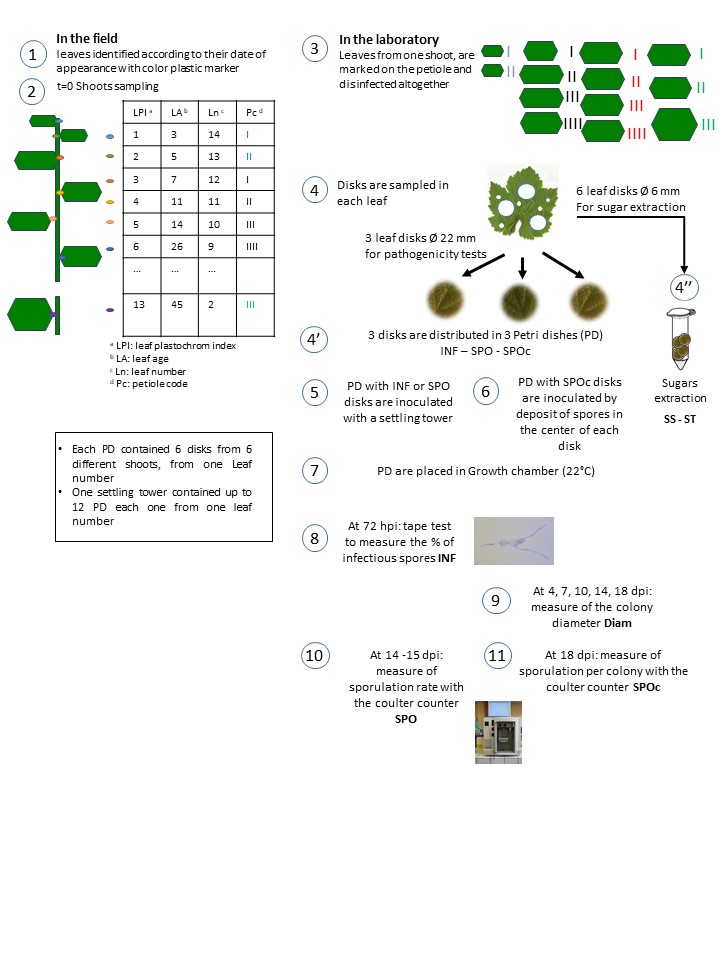

Supplement: Supplementary Figure S1 — Scheme of the experimental design. [file Image_1.JPEG]
